# 

**Published:** 1966-04

**Authors:** 


### BRISTOL MEDICO-CHIRURGICAL JOURNAL

*(Incorporating the Medical Journal of the South-West)*

Established 1883

VOL. 81 (ii) April 1966 NO. 300

### Contents

TUMOURS OF THE SKIN *D. C. Bodenham* 23

SEROLOGICAL TESTS FOR SYPHILIS *H. R. Cayton* 32

THE BIOCHEMICAL DETECTION OF INHERITED DISEASES *J. B. Holton* 35

NOTES AND NEWS . . . . . . . . . . . . . 40

*PUBLISHED QUARTERLY*

*ANNUAL SUBSCRIPTION* **16/-** *POST FREE*

BRISTOL MEDICO-CHIRURGICAL JOURNAL
PUBLISHED UNDER THE AUSPICES OF THE

### *Bristol Medico-Chirurgical Society*

The Society was founded in 1874. In 1883 publication of this Journal began, an has continued quarterly since then. During the years 1949 to 1962 it appeared und the title of "The Medical Journal of the South West".

The Society founded a medical library in 1890, which in 1892 was combined wi that of the Bristol Medical School. This became the University of Bristol Medic Library in 1925, and an agreement in that year confirmed the privilege of Members use the University Medical Library.

---

### *Editorial Committee*

Dr. N. J. Brown, Southmead Hospital, Bristol. *Joint Editor.*

Dr. A. B. Raper, Department of Pathology, Bristol Royal Infirmary. *Joint Editor.*

Dr. J. Macrae, Ham Green Hospital.

Dr. R. C. Wofinden, Central Clinic, Bristol.

Mr. A. E. S. Roberts, Medical Library, University of Bristol. *Secretary.*

*Local Correspondents*

*Bath* . . Mr. A. D. Bateman, 3 The Circus, Bath.

*Exeter* . . Dr. L. N. Jackson, Mount Jocelyn, Crediton, Devon.

*Gloucester* . Dr. R. F. Jarrett, 9 College Green, Gloucester.

*Plymouth* . Dr. C. R. Croft, Lexden, Hartley Av., Plymouth.

*Taunton* . Dr. M. McAlley, 1 The Crescent, Taunton.

*Torquay* . Dr. A. Robinson Thomas, 20 Devon Square, Newton Abbot, Devon.

*Truro* . . Dr. F. D. M. Hocking, St. Andrews, Perranporth, Cornwall.

*Yeovil* . . Mr. J. A. Vere Nicoll, Knapp House, Yeovil, Somerset.

### NOTICE TO CONTRIBUTORS

Original articles are invited, provided they have not been published elsewhere. Notes of forthcoming events, meetings held, degrees conferred, staff appointments, and other local news are welcomed. The editorial committee reserves the right to make literary corrections.

Illustrations should always be supplied ready for reproduction. All re-touchings or other operations required will be charged to the author. Line drawings should be drawn in Indian ink. We regret that we must ask authors to pay for making blocks, if this is necessary.

J. W. ARROWSMITH LTD., WINTERSTOKE ROAD
BRISTOL.

### *Notes and News*

An honorary fellowship of the Royal College of Surgeons of England has been conferred on Sir Philip Morris.

### UNIVERSITY OF BRISTOL

*Examination Results*

*M.D.:* J. Brunning, A. R. Daniel, J. James, J. Little, June Lloyd.

*Ph.D.:* B. J. Alps, R. J. Andlaw, Vanda M. Lucke, N. R. Thomas.

*Ch.M.:* D. N. Walder.

*Printed by*
J. W. Arrowsmith Ltd., Bristol

